# Oligonucleotide Based Magnetic Bead Capture of *Onchocerca volvulus* DNA for PCR Pool Screening of Vector Black Flies

**DOI:** 10.1371/journal.pntd.0001712

**Published:** 2012-06-19

**Authors:** Hemavathi Gopal, Hassan K. Hassan, Mario A. Rodríguez-Pérez, Laurent D. Toé, Sara Lustigman, Thomas R. Unnasch

**Affiliations:** 1 Centro de Biotecnología Genómica, Instituto Politécnico Nacional, Reynosa, Tamaulipas, México; 2 Department of Global Health, Global Health Infectious Disease Research Program, University of South Florida, Tampa, Florida, United States of America; 3 Multi-Disease Surveillance Centre, Ouagadougou, Burkina Faso; 4 Laboratory of Molecular Parasitology, Lindsley F. Kimball Research Institute, New York Blood Center, New York, New York, United States of America; University of Pittsburgh, United States of America

## Abstract

**Background:**

Entomological surveys of *Simulium* vectors are an important component in the criteria used to determine if *Onchocerca volvulus* transmission has been interrupted and if focal elimination of the parasite has been achieved. However, because infection in the vector population is quite rare in areas where control has succeeded, large numbers of flies need to be examined to certify transmission interruption. Currently, this is accomplished through PCR pool screening of large numbers of flies. The efficiency of this process is limited by the size of the pools that may be screened, which is in turn determined by the constraints imposed by the biochemistry of the assay. The current method of DNA purification from pools of vector black flies relies upon silica adsorption. This method can be applied to screen pools containing a maximum of 50 individuals (from the Latin American vectors) or 100 individuals (from the African vectors).

**Methodology/Principal Findings:**

We have evaluated an alternative method of DNA purification for pool screening of black flies which relies upon oligonucleotide capture of *Onchocerca volvulus* genomic DNA from homogenates prepared from pools of Latin American and African vectors. The oligonucleotide capture assay was shown to reliably detect one *O. volvulus* infective larva in pools containing 200 African or Latin American flies, representing a two-four fold improvement over the conventional assay. The capture assay requires an equivalent amount of technical time to conduct as the conventional assay, resulting in a two-four fold reduction in labor costs per insect assayed and reduces reagent costs to $3.81 per pool of 200 flies, or less than $0.02 per insect assayed.

**Conclusions/Significance:**

The oligonucleotide capture assay represents a substantial improvement in the procedure used to detect parasite prevalence in the vector population, a major metric employed in the process of certifying the elimination of onchocerciasis.

## Author Summary

The absence of infective larvae of *Onchocerca volvulus* in the black fly vector of this parasite is a major criterion used to certify that transmission has been eliminated in a focus. This process requires screening large numbers of flies. Currently, this is accomplished by screening pools of flies using a PCR-based assay. The number of flies that may be included in each pool is currently limited by the DNA purification process to 50 flies for Latin American vectors and 100 flies for African vectors. Here, we describe a new method for DNA purification that relies upon a specific oligonucleotide to capture and immobilize the parasite DNA on a magnetic bead. This method permits the reliable detection of a single infective larva of *O. volvulus* in pools containing up to 200 individual flies. The method described here will dramatically improve the efficiency of pool screening of vector black flies, making the process of elimination certification easier and less expensive to implement.

## Introduction

Onchocerciasis, or river blindness has historically represented one of the most important neglected tropical diseases in the developing world as measured as a cause of socio-economic disruption [1]. It is also considered a candidate for elimination by the international community [2], [3]. As a result of these factors, the international community is currently supporting several programs whose goals are either to eliminate the disease as a public health problem, or to locally eliminate the causative agent of the disease, *Onchocerca volvulus*. These include the Onchocerciasis Elimination of the Americas (OEPA), the African Programme for Onchocerciasis Control (APOC), and the Ugandan Onchocerciasis Elimination Program (UOEP).

Entomological criteria play an important role in the elimination criteria recommended by the World Health Organization (WHO) [4] and those currently utilized by OEPA [5]. Entomological data play an especially important role in the certification of elimination following the cessation of treatment, as the prevalence of infective stages of the parasite in the fly population is the timeliest measure of transmission in a given area. However, demonstrating that transmission is interrupted requires that large numbers of flies be tested. For example, current OEPA guidelines require that the prevalence of flies carrying infective larvae (L3) be less than 1/2000 in every sentinel community for transmission to be interrupted [5]. In order to be able to state with a 95% confidence that the prevalence of infective flies is less that 1/2000 requires examining approximately 6000 flies from each sentinel community. Examining such large numbers of insects using conventional methods (dissection) is impractical. For this reason, the current guidelines recommend the use of pool screening PCR based methods to conduct the entomological studies necessary to document transmission interruption [4].

Currently, the accepted method for pool screening vector black flies to detect *O. volvulus* relies upon screening DNA prepared from fly pools with a PCR assay targeting a repeated sequence family (the O-150 repeat [6]) specific for parasites of the genus *Onchocerca*. Algorithms have been developed that permit one to derive a point estimate of the prevalence of infection in the fly population (and an associated confidence interval) from the number of PCR positive pools and the number of flies contained in each pool [7]. Furthermore, because the infective stage of the parasite is the only form found in the black fly head capsule, separated pools of heads and bodies may be screened to obtain estimates of the prevalence of infective flies (flies with infective stages in their head) and the prevalence of infected flies (flies with immature larval stages in their bodies). This approach has been used to monitor transmission of *O. volvulus* in many foci of Latin America and Africa [8]–[11], as well as to certify the interruption of transmission in foci on both continents [5], [12]–[14].

Previous modeling studies have shown that increasing pool sizes has relatively little effect on the accuracy of the estimate of prevalence of infection obtained, so long as the proportion of positive pools remains less than the majority of pools screened [15]. Thus, in situations where pool screening is used to certify transmission interruption (where infective flies are extremely rare or non-existent) the pool size is only limited by the biochemical constraints of the assay. The current method of DNA extraction for the O-150 PCR assay is based upon adsorption to a silica matrix [16]. This preparation results in DNA samples that still contain inhibitors of the PCR, limiting the number of flies that may be included in each pool. Currently, pool sizes are limited to 50 individual heads or bodies (in the case of flies from Latin America) [9] or 100 individual heads or bodies (in the case of flies from Africa) [7]. Developing alternative methods to prepare DNA that would permit an increase in the maximum number of heads or bodies in each pool would decrease the cost and effort necessary to screen the requisite large numbers of flies necessary to certify transmission interruption.

Magnetic bead based purification protocols have been developed for many different pathogens. Most of these involve direct capture of the pathogen using beads coated with pathogen-specific antibodies. This method, known as immunomagnetic separation (IMS), has been successfully used to purify and concentrate viruses [17], bacteria [18], [19] and fungal [20] pathogens. Similarly, methods have been developed which use oligonucleotides to magnetically purify pathogen genomic DNA [21].

Here, we describe a magnetic bead capture method to isolate *O. volvulus* DNA from homogenates prepared from pools of Latin American and African *Simulium* vector black flies. This method is shown to be an improvement upon the current DNA purification method utilizing organic extraction and silica adsorption.

## Materials and Methods

### Black flies and *O. volvulus* infective larvae

*Simulium ochraceum* s.l. females were collected in public areas of the community of José María Morelos y Pavón, Chiapas, México between the hours of 0700 and 1000. Previous studies have demonstrated that the majority of flies captured during this period were nulliparous, and the risk of infection was therefore minimal.

*Simulium damnosum* s.l. were obtained from breeding sites on public land located near the communities of Bodajugu and Sakora. These communities are located in the Region des Cascades in Southwestern Burkina Faso. This region is located within the area of the former Onchocerciasis Control Programme in West Africa, where onchocerciasis has been eliminated as a public health problem.

*O. volvulus* L3 were obtained from experimentally infected *Simulium damnosum* s.l. flies 7 days after infection with skin microfilariae. The flies were kept at 25°C and 80% relative humidity to allow the microfilariae to develop into L3. Larvae were isolated from the flies by dissection into dissecting medium (IMDM+10% FCS+2x Penicillin-Streptomycin-Fungizone) using a dissecting microscope. The cleaned larvae were frozen in 9% DMSO, 4 mM PVP, 10% FCS in Grace medium using Bio-Coll (freezing to −40°C at 1°C/minute followed by 30 minutes at −40°C) and then transferred to liquid nitrogen for long-term storage. The parasite material was prepared in the Tropical Medicine Research Station, Kumba, Cameroon, and is being stored at the New York Blood Center.

### DNA extraction by adsorption to a silica matrix

Pools containing varying numbers of black flies were prepared and the heads and bodies separated by freezing and agitation, as previously described [7]. A single *O. volvulus* L3 was added to each pool. Head and body pools were placed in a 1.5 ml microcentrifuge tube and purified using magnetic silica coated beads (Machery-Nagel GmbH & Co, Bethlehem, PA, USA) following the instructions provided by the manufacturer. In brief, the pools were homogenized in 200 µl of T1 buffer, 25 µl of proteinase K solution provided in the kit was added and the homogenates were incubated at 56°C for 30 minutes. The homogenates were subjected to centrifugation at 13,400×g for 5 minutes at room temperature. A total of 225 µl of the supernatant was transferred to a fresh tube containing 24 µl B-beads (Machery-Nagel) and 360 µl MB2 buffer (Machery-Nagel), and the tube shaken for 5 minutes at room temperature. The magnetic beads were isolated by placing the tubes in a six tube magnetic separator (Dynal MPC-S; Invitrogen). The supernatants were removed and discarded, and 600 µl of MB3 wash buffer was added to each sample. The bead/DNA complexes were washed by shaking for 5 min at room temperature. The beads were collected in the magnetic separator as before, and the washing procedure repeated with successive washes with 600 µl of MB4 and MB5 wash buffers (Machery-Nagel). Following the wash in the MP5 buffer, the beads were exposed to air for one minute to permit the traces of ethanol to evaporate. DNA was eluted from the beads by the addition of 100 µl of elution buffer. The beads were shaken for 5 minutes at room temperature to elute the DNA, and the beads removed by placing the tubes in the magnetic separator. The supernatant containing the purified DNA was then transferred to a fresh tube.

### Oligonucleotide capture of *O. volvulus* DNA

Pools of spiked heads and bodies were prepared as described above. The head and body pools were homogenized in 500 µl of 10 mM Tris-HCl (pH 8.0) 1 mM EDTA, and proteinase K added to a final concentration of 2 mg/ml. The homogenates were incubated at 56°C for 2 hours, and dithiothreitol added to a final concentration of 20 mM. The samples were heated to 100°C for 30 minutes and subjected to three freeze-thaw cycles. The homogenates were subjected to centrifugation at 13,400×g for 5 minutes and the supernatant placed into a new tube. The solutions were brought to a final concentration of 100 mM Tris-HCl (pH 7.5) 100 mM NaCl. A total of 5 µl of a 0.5 µM solution of OVS2-biotin primer (5′B-AATCTCAAAAAACGGGTACATA-3′, where B = biotin) was added to each sample. The samples were then heated to 95°C for three minutes and allowed to cool slowly to room temperature.

While the probe was annealing to the DNA in the solution, 10 µl of Dynal M-280 strepavidin coated beads (Invitrogen) were placed in a single well of a 96 well tissue culture plate. The plate was placed on a magnetic capture unit (Dynal MPC-96, Invitrogen) and the beads collected for 2 minutes. The beads were then washed five times with 200 µl binding buffer (100 mM Tris-HCl (pH 7.5) 100 mM NaCl) per wash, resuspended in 10 ul and added to the sample. The samples were incubated at 4°C overnight on a roller to permit the oligonucleotide-DNA hybrids to bind to the beads. The samples were placed in the magnetic separator for two minutes to capture the beads and the supernatant discarded. The beads were resuspended in 150 µl of binding buffer by pipetting, and the beads captured by placing the tubes in the magnetic separator for two minutes. The wash step was repeated five times. The beads were then resuspended in 20 µl of sterile water, heated to 80°C for 2 minutes and cooled rapidly on ice for two minutes. The beads were removed by placing the tubes in the magnetic capture apparatus, and the supernatant containing the purified DNA transferred to a new tube.

### O-150 PCR analysis

A total of 2.5 µl of the purified genomic DNA was used as a template for the PCR amplifications carried out in a total volume of 50 µL containing 0.5 µmol/L of O-150 primer (5′-GATTYTTCCGRCGAANARCGC-3′) and 0.5 µmol/L of biotinylated O-150 primer (5′-B-GCNRTRTAAATNTGNAAATTC- 3′, where B = biotin; N = A, G, C, or T; Y = C or T; and R = A or G). Reaction mixtures also contained 60 mM Tris-HCl, (pH 9.0), 15 mM (NH4)_2_SO4, 2 mM MgCl_2_, 0.2 mM each of dATP, dCTP, dGTP and dTTP, and 2.5 units of Taq polymerase (Invitrogen). Cycling conditions consisted of five cycles of one minute at 94°C, two minutes at 37°C, and 30 seconds at 72°C, followed by 35 cycles of 30 seconds each at 94°C , 37°C , and 72°C. The reaction was completed by incubating at 72°C for six minutes.

Amplification products were detected by PCR enzyme-linked immunosorbent assay (ELISA), essentially as previously described [10]. Briefly, 5 µl of each PCR reaction was bound to a streptavidin-coated ELISA plate, and the DNA strands denatured by treatment with alkali. The bound PCR fragments were then hybridized to a fluorescein-labeled *O. volvulus*-specific oligonucleotide probe (OVS2: 5′-AATCTCAAAAAACGGGTACATA-FL-3′), and the bound probe detected with an alkaline phosphatase-labeled anti-fluorescein antibody (fragment FA; Roche Diagnostics). Bound antibody was detected using the ELISA amplification reagent (BluePhos) kit from KPL (Gaithersburg, USA) following the manufacturer's instructions. Color development was stopped by the addition of 100 µl AP stop solution, and the plates read in an ELISA plate reader set at 630 nm. Samples were scored positive if their optical density exceeded either the mean plus three standard deviations of ten negative control wells run in parallel or 0.1, whichever was greater.

## Results

An initial series of experiments were carried out with pools of heads and bodies of *S. ochraceum* s.l. (a major Latin American vector of onchocerciasis). Pools containing varying numbers of heads of bodies were spiked with a single *O. volvulus* L3, and DNA prepared from the pools using either the conventional method of organic extractions followed by adsorption to a silica matrix, or by oligonucleotide capture of *O. volvulus* genomic DNA followed by magnetic purification of the captured oligonucleotide-DNA complexes. The conventional method consistently produced a positive signal in pools containing up to 50 heads (Table 1). Pools containing greater than 50 heads were not positive in the assay. All samples containing 50, 100 or 150 *S. ochraceum* s.l. bodies were positive, while none of the pools containing 200 bodies were positive (Table 1). In contrast, positive signals were obtained in all pools containing up to 200 heads or bodies in the assays performed on the oligonucleotide capture purified DNA samples (Table 1).

**Table 1:** Performance of silica adsorption and oligonucleotide capture methods with different size pools of *S. ochraceum*.

Pool size	Sample	Silica Positive	Mean Silica ELISA OD (range)*	Capture positive	Mean Capture ELISA OD (range)*
50	Heads	100% (3/3)	0.286 (0.142–0.476)	100% (3/3)	0.647 (0.637–0.667)
100	Heads	0% (0/3)	0.088 (0.071–0.098)	100% (3/3)	0.662 (0.659–0.667)
150	Heads	0% (0/3)	0.082 (0.076–0.094)	100% (3/3)	0.638 (0.630–0.645)
200	Heads	0% (0/3)	0.080 (0.078–0.081)	100% (3/3)	0.603 (0.600–0.608)
50	Bodies	100% (3/3)	0.119 (0.104–0.134)	100% (3/3)	0.113 (0.110–0.119)
100	Bodies	100% (3/3)	0.116 (0.104–0.127)	100% (3/3)	0.192 (0.190–0.193)
150	Bodies	100% (3/3)	0.120 (0.104–0.149)	100% (3/3)	0.261 (0.260–0.262)
200	Bodies	0% (0/3)	0.071 (0.062–0.081)	100% (3/3)	0.231 (0.230–0.232)

*: mean of positive control wells = 0.431 (range 0.369–0.492).

The preliminary experiments suggested that the oligonucleotide capture method was capable of detecting one L3 in pools of up to 200 heads or bodies. To further explore the sensitivity of the assay, the experiment was repeated using 10 separate pools containing 200 heads or bodies spiked with a single L3. All pools were found to be positive, suggesting that the oligonucleotide capture assay was capable of consistently detecting a single L3 in pools of up to 200 heads or bodies of *S. ochraceum* s.l. (Table 2).

**Table 2:** Performance of oligonucleotide capture purified DNA on pools of 200 flies.

Species	Pool size	Sample type	% positive	Mean ELISA OD (range)*
*S. ochraceum*	200	Heads	100% (10/10)	0.347 (0.146–0.762)
*S. ochraceum*	200	Bodies	100% (10/10)	0.275 (0.144–0.599)
*S. damnosum*	200	Heads	100% (5/5)	0.772 (0.667–0.822)
*S. damnosum*	200	Bodies	100% (5/5)	0.302 (0.183–0.380)

*: mean of positive control wells = 0.526 (range 0.410–0.686).

Previous studies had demonstrated that the conventional silica adsorption method was capable of detecting a single infected *S. damnosum* s.l. (the major African vector of onchocerciasis) fly in a pool containing up to 99 uninfected flies [7]. To determine if the performance of the oligonucleotide capture assay was similar when applied to *S. damnosum* s.l., the spiking experiments were repeated employing pools containing 200 *S. damnosum s.l.* heads or bodies. All spiked pools were found to be positive (Table 2), suggesting that the capture assay preformed equally well on both African and Latin American vectors of onchocerciasis.

For the capture assay to be cost effective, it should be competitive with the cost of the conventional silica adsorption assay. The two assays require roughly the same amount of technical time, so labor costs may be assumed to be equivalent per sample for the two assays. However, because it will be possible to increase the size of the pools 2–4 fold when using the capture assay, a reduction in labor costs of between 50% and 75% would be realized when costs are considered on a per-fly-tested basis. Similarly, the per-sample cost of carrying out the conventional assay is roughly $2.22 per pool, while the cost of the magnetic bead assay is $3.81 per pool. However, because the magnetic bead permits more flies to be tested per pool, cost savings in reagents are realized when the costs are amortized on a per fly basis (Table 3).

**Table 3:** Cost analysis of silica adsorption and oligonucleotide capture assays.

Step	Traditional (pool size 50)	Traditional (pool size 100)	Oligonucleotide capture
DNA purification	6.08	3.04	3.11
PCR	2.36	1.18	0.59
ELISA detection	0.45	0.225	0.11
TOTAL	8.89	4.445	3.81

Costs are expressed as the cost to analyze 200 individual flies, in US dollars.

## Discussion

Previous studies have demonstrated that the algorithms used to predict the prevalence of infection in a population from data derived from screening pools of samples are relatively insensitive to the size of the pool, so long as the proportion of positive pools does not represent a substantial majority of the samples screened [15]. Thus, the size of pools used in a pool screening protocol is more likely to be limited by the biochemistry of the detection assay than by the underlying statistical uncertainties associated with screening pools of samples. This is particularly true when positive samples are extremely rare, as in the case when monitoring for transmission interruption. The data presented above suggest that the oligonucleotide capture method of purifying *O. volvulus* DNA is superior to the conventional silica adsorption method. Mixing experiments have demonstrated that PCR inhibitors carried through the silica adsorption process limits the size of the pools that may be screened to 50 individual flies for *S. ochraceum* and 100 for *S. damnosum s.l* (data not shown). The oligonucleotide capture method appears to result in DNA preparations that are freer of PCR inhibitors than are those prepared using silica adsorption. The practical result of this improvement is that it permits a 2 to 4-fold increase in the number of black fly heads or bodies that can be included in a single pool. This increase in pool size results in a dramatic cost savings in the per-unit cost of the O-150 pool screen assay. Using the oligonucleotide capture assay, labor costs are reduced by 50–75%, while the overall cost of reagents needed is $3.81 per pool of 200 flies or less than $0.02 per individual fly. The decrease in cost and corresponding increase in the efficiency of the assay will make it more practical to screen the large numbers of flies necessary to demonstrate transmission interruption and to certify elimination of onchocerciasis.

Because the oligonucleotide capture assay is a modification of the conventional silica adsorption assay, the equipment required to carry out both assays is quite similar. The only additional equipment necessary when replacing the conventional assay with the oligonucleotide assay is the magnetic capture apparatus. This unit is relatively inexpensive, costing less than $579 (Invitrogen's list price). This would be recovered in reagent costs alone after screening just 114 pools of flies.

The O-150 PCR is based upon the amplification of a genus specific tandemly repeated DNA sequence present in the genome of *Onchocerca* parasites [22]. Thus, the standard O-150 PCR will amplify sequences present in all *Onchocerca* species, including *Onchocerca ochengi*, a cattle parasite that is sympatric with *O. volvulus* in sub-Saharan Africa. Currently, the O-150 PCR is made species or strain specific by modifying the amplification conditions to limit amplification to species specific members of the O-150 repeat family [23] or by the use of species or strain specific probes to detect the resulting amplicons [8]. However the oligonucleotide used in the capture assay (OVS2) has previously been shown to hybridize specifically to *O. volvulus* under appropriate conditions [22]. It is therefore possible that modification of the current capture conditions could result in a purification process that would specifically capture *O. volvulus* but not *O. ochengi* genomic DNA. This would provide an extra layer of specificity to the assay when it is employed in areas where *O. volvulus* and *O. ochengi* are sympatric.
